# *In vivo* imaging of tau pathology using multi-parametric quantitative MRI

**DOI:** 10.1016/j.neuroimage.2015.02.023

**Published:** 2015-05-01

**Authors:** J.A. Wells, J.M. O'Callaghan, H.E. Holmes, N.M. Powell, R.A. Johnson, B. Siow, F. Torrealdea, O. Ismail, S. Walker-Samuel, X. Golay, M. Rega, S. Richardson, M. Modat, M.J. Cardoso, S. Ourselin, A.J. Schwarz, Z. Ahmed, T.K. Murray, M.J. O'Neill, E.C. Collins, N. Colgan, M.F. Lythgoe

**Affiliations:** a UCL Centre for Advanced Biomedical Imaging, Division of Medicine and Institute of Child Health, University College London, UK; b Brain Repair & Rehabilitation, Institute of Neurology, University College London, UK; c Translational Imaging Group, Centre for Medical Imaging Computing, University College London, UK; d Eli Lilly & Co. Ltd, Erl Wood Manor, Windlesham, Surrey GU20 6PH, UK; e Eli Lilly and Company, Lilly Corporate Center, Indianapolis, IN 46285, USA

## Abstract

As the number of people diagnosed with Alzheimer's disease (AD) reaches epidemic proportions, there is an urgent need to develop effective treatment strategies to tackle the social and economic costs of this fatal condition. Dozens of candidate therapeutics are currently being tested in clinical trials, and compounds targeting the aberrant accumulation of tau proteins into neurofibrillary tangles (NFTs) are the focus of substantial current interest. Reliable, translatable biomarkers sensitive to both tau pathology and its modulation by treatment along with animal models that faithfully reflect aspects of the human disease are urgently required. Magnetic resonance imaging (MRI) is well established as a valuable tool for monitoring the structural brain changes that accompany AD progression. However the descent into dementia is not defined by macroscopic brain matter loss alone: non-invasive imaging measurements sensitive to protein accumulation, white matter integrity and cerebral haemodynamics probe distinct aspects of AD pathophysiology and may serve as superior biomarkers for assessing drug efficacy. Here we employ a multi-parametric array of five translatable MRI techniques to characterise the *in vivo* pathophysiological phenotype of the rTg4510 mouse model of tauopathy (structural imaging, diffusion tensor imaging (DTI), arterial spin labelling (ASL), chemical exchange saturation transfer (CEST) and glucose CEST). Tau-induced pathological changes included grey matter atrophy, increased radial diffusivity in the white matter, decreased amide proton transfer and hyperperfusion. We demonstrate that the above markers unambiguously discriminate between the transgenic group and age-matched controls and provide a comprehensive profile of the multifaceted neuropathological processes underlying the rTg4510 model. Furthermore, we show that ASL and DTI techniques offer heightened sensitivity to processes believed to precede detectable structural changes and, as such, provides a platform for the study of disease mechanisms and therapeutic intervention.

## Highlights

• The rTg4510 mouse model exhibits specific tau pathology.
• This enables dissection of the unique role of tau in Alzheimer's Disease.
• We applied five quantitative, non-invasive MRI techniques to the rTg4510.
• We demonstrate the sensitivity of each to discriminate regions of high tau burden.
• This study provides a platform for the longitudinal assessment of tau therapies.

## Introduction

Alzheimer's disease (AD) is a continuum of relentless irreversible biochemical and pathological changes that lead to pronounced neurodegeneration. Factors implicated in the multifaceted pathogenesis include local inflammation (Akiyama et al., May-Jun, 2000), protein accumulation (Fletcher et al., 2013), abnormal mitochondrial function (Maruszak and Zekanowski, 2011) and haemodynamic alterations (de la Torre, 2002) with amyloid plaques and neurofibrillary tangles (NFTs) being the classical hallmarks of the post-mortem AD brain. Currently the most established methods for the biological assessment of disease progression in patients are estimation of regional brain atrophy using structural MRI, PET imaging of glucose metabolism using fluorodeoxyglucose (FDG) and imaging of amyloid deposits (e.g. using [^11^C]-PIB or [^18^F]-Florbetapir) as well as invasive CSF sampling to measure tau and amyloid moieties (Langbaum et al., 2013), all of which target specific components of the pathobiological cascade. However, other detectable features of AD progression may exist that have yet to be fully explored as potential imaging biomarkers and as such may provide greater mechanistic insight into disease progression and improve the scope of evaluation of emerging therapies. In this work we sought to investigate new biomarkers of AD, sensitive to specific tissue properties: perfusion, microstructure, chemical exchange and glucose uptake. Specifically, we implemented the novel chemical exchange saturation transfer (CEST) MRI for targeted detection of impeded proton exchange that may occur due to the presence of aggregated protein. The CEST method saturates mobile protons of the macromolecular pool that are exchanging with the bulk water pool (for recent reviews see Liu et al., 2013, van Zijl and Yadav, 2011). In this study we used CEST to probe amide -proton transfer in grey matter structures. We also implemented diffusion tensor imaging (DTI) (a sensitive measure of local tissue inflammation and cellular integrity) to probe changes in microstructural compartmentation. Finally we applied arterial spin labelling (ASL) and glucose CEST (glucoCEST) to measure perfusion and glucose delivery and uptake to infer changes in cerebral metabolism, vasculature and neuronal excitability and compare these measures to the more established structural MRI biomarkers and histology.

Transgenic mouse models are increasingly being used to further our knowledge of the cause and progression of AD, and identify new targets for therapeutic intervention (Chin, 2011, Games et al., 1995, Ramsden et al., 2005, Spires et al., 2005). While no mouse recapitulates all characteristics of the human condition, such models do permit the study of specific pathological features in experimentally controlled conditions. This is particularly advantageous in AD where human clinical research can often be confounded by uncontrollable variables such as lifestyle choices and other co-morbidity; mice allow the precise impact of genes and their role in AD to be fully explored. Mice containing mutations in the amyloid precursor protein (APP) gene are the oldest and most widely studied models of AD, and are used to investigate the role of APP, amyloid-beta and amyloidosis in neurodegeneration (Hall and Robertson, 2012). APP and presenilin transgenic mice are also valuable tools in preclinical drug discovery, where many of the emerging AD treatments focus on decreasing amyloid-beta production and aggregation. However, while several of these compounds have shown promising preclinical results, they have had little to no success in clinical trials. This may in part be due to the fact that while most APP mice form plaques they do not have tangle pathology or evidence of robust neurodegeneration. The failure of these candidate drugs to date to show any improvements in the human condition has caused a shift in interest to other therapeutic pathways (Clavaguera et al., 2009). In recent years, tau has emerged as a potential target for therapeutic intervention. Tau plays a critical role in the neurodegenerative process: aggregates of hyperphosphorlylated tau forming neurofibrillary tangles (NFTs) are a major hallmark of AD and correlate with clinical disease progression (Braak and Braak, 1991). In the absence of amyloid-beta, NFTs are also implicated in several other neurodegenerative diseases (frontotemporal dementia with parkinsonism, progressive supranuclear palsey, corticobasal degeneration), further supporting the role of tau in the dementing process (Lee et al., 2001). A mouse model of tauopathy (rTg4510) has been developed that specifically expresses tau in the forebrain but without amyloid plaques, enabling dissection of the unique role of tau in AD pathology (Ramsden et al., 2005, Santacruz et al., 2005). Behavioural and histological studies of the rTg4510 mouse have demonstrated cognitive deficits in learning and motor tasks and marked atrophy of brain regions known to be selectively vulnerable to AD such as the hippocampus and frontal cortex (Santacruz et al., 2005).

In this study, we target diverse and specific components of tau pathology by applying multi-parametric MRI to the rTg4510 model. We show that each of the MRI methods can unambiguously discriminate tau pathology from healthy control subjects, providing a platform for the longitudinal assessment of experimental treatments using non-invasive imaging techniques. Furthermore, we demonstrate that ASL and DTI metrics are sensitive to tau-pathology in regions of low NFT density prior to significant atrophy, providing evidence that these data may inform the development of a multi-parametric imaging biomarker for early detection of tau pathology in the clinic. This is the first application of ASL and CEST to the rTg4510 model as well as the first report of glucoCEST to investigate neurodegenerative disease.

## Materials and methods

### Transgenic mice

Generation of homozygous rTg4510 transgenic mice has been reported previously (Ramsden et al., 2005). Female rTg4510 mice were licensed from the Mayo Clinic (Jacksonville Florida, USA) and bred for Eli Lilly by Taconic (Germantown, USA). Mice were imported to the UK for imaging studies at the Centre for Advanced Biomedical Imaging (CABI), London. All studies were carried out in accordance with the United Kingdom Animals (Scientific Procedures) act of 1986.

### Magnetic resonance imaging

All imaging was performed with a 9.4 T VNMRS horizontal bore scanner (Agilent Inc.). A 72 mm inner diameter volume coil (Rapid Biomedical) was used for RF transmission and signal was received using a 4 channel array head coil (Rapid Biomedical). Mice were anaesthetised under 2% isoflurane and positioned in a MRI compatible head holder to minimise motion artefacts. Anaesthesia was then maintained at 1.5% isoflurane in 100% O_2_ throughout imaging. Core temperature and respiration were monitored using a rectal probe and pressure pad (SA instruments). Mice were maintained at ~ 37 °C using heated water tubing and a warm air blower with a feedback system (SA instruments).

In this study, two separate cohorts of both rTg4510 and wild-type litter matched control mice at 8.5 and 9.5 months of age were imaged. In the first cohort (8.5 months) we conducted ASL, DTI and 3D structural imaging to N = 9 rTg4510 mice and N = 17 wild-type controls. The brains of each of the rTg4510 and wild-type mice were perfused fixed for histology directly after imaging (see Perfuse fixation). In the second cohort (9.5 months) we acquired CEST data from N = 10 rTg4510 and N = 9 WT mice and gluco-CEST data from N = 5 rTg4510 and N = 5 wild-type control mice that were fasted for 24 h prior to the imaging experiment. The reported p-values are from standard t-tests for differences in parameters between the rTg4510 and WT groups, unless stated otherwise.

### Structural imaging and processing for Tensor Based Morphometry (TBM)

A 3D T2-weighted sequence was employed for structural imaging with parameters: FOV = 19.2 mm × 16.8 mm × 12.0 mm; resolution = 150 μm × 150 μm × 150 μm; TR = 2500 ms, TEeff = 43 ms, ETL = 4, NSA = 1. In order to estimate the volume of the cortex and hippocampus, ROIs were delineated using open source software (ITK-SNAP). ROIs across all slices containing the hippocampus were manually drawn by the same expert with visual reference to a mouse brain atlas.

For TBM analysis, *in*-*vivo* structural images were automatically oriented to a standard atlas space (Right Antero-Superior), corrected for intensity non-uniformity using the N3 algorithm (Sled et al., 1998), and skull stripped using a STAPLE algorithm (Cardoso et al.,2013) to combine masks from several prior atlases registered to the data. Intensities were normalised (Nyul et al., 2000) and a multi-iteration group-wise registration (implemented in the open-source *NiftyReg* software Modat et al., 2010) was performed as follows to align equivalent voxels between subjects. First, all subjects were rigidly aligned to a randomly-chosen target member of the group. This was followed by four iterations of affine registration (12 degrees of freedom), using a block-matching algorithm based upon normalised mutual information, and 20 iterations of non-rigid registration (NRR), based upon symmetric free-form deformation. After each iteration, the intensity average image was found and used as the target for the subsequent registration. Deformation fields were transformed by taking the log of the determinant of the Jacobian matrix calculated at each voxel, to give that voxel's relative expansion or contraction in the final average image compared to each original. These values were smoothed with a 0.2 mm FWHM Gaussian kernel to account for registration error and to render the values closer to a normal distribution, and mass-univariate statistics (two-tailed t-tests) were performed on each voxel, fitting a General Linear model controlling for total intracranial volume (TIV) based upon the brain masks. The resulting statistical parametric map was corrected for multiple tests using the False Discovery Rate (FDR (Genovese et al., 2002), q = 0.05). We applied a cluster size threshold of 20 voxels.

### Arterial spin labelling (ASL) to image cerebral perfusion

**Fig. 1:**
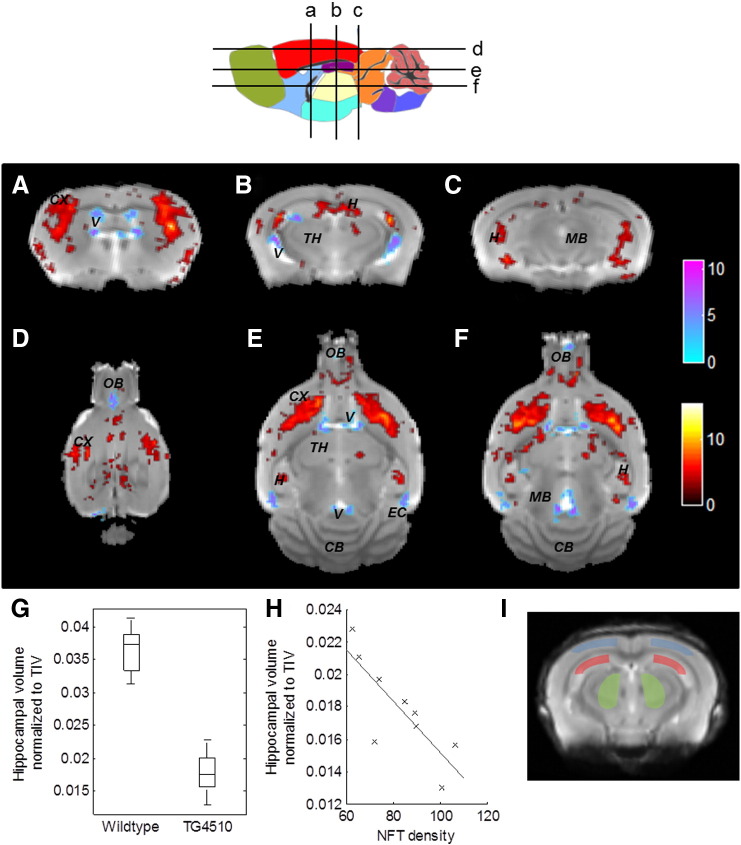
High resolution structural MRI. A–F) Results from structural analysis, showing TBM statistical results through representative coronal and axial slices of the final average image after 20 iterations of NRR (locations indicated on schematic diagram above). Red: TG4510 brains relatively locally smaller than the average; blue: larger. Based upon FDR-corrected t-statistics (q = 0.05), controlling for total intracranial volume. Clusters smaller than 20 voxels were removed. CB: cerebellum; CX: cortex; EC: entorhinal cortex; H: hippocampus; MB: midbrain; OB: olfactory bulbs; TH: thalamus; V: Ventricles. G) Manually segmented hippocampal volumes (WT and TG), boxplots report median, interquartile range and range. H) Comparison of manually segmented hippocampul volume (rTg4510) to PG positive NFT density, where a significant negative correlation was observed (Pearson's correlation coefficient = 0.81, p = 0.01). I) Typical manual grey matter ROIs used to report average ASL, DTI, CEST, GlucoCEST from the cortex (blue), hippocampus (red) and thalamus (green), overlaid on the average image.

A flow-sensitive alternating inversion recovery (FAIR) sequence (Kim, 1995, Kwong et al., 1992) with a 4-shot segmented spin-echo echo-planar imaging (EPI) readout was implimented with the following parameters: 5 slices, slice thickness = 1 mm, FOV = 20 × 20 mm, matrix size = 64 × 64, slice selective inversion pulse width = 12 mm, 5 inversion times (TI = 600, 1250, 1500, 2000, 2500 ms), TE = 11 ms, TR = TI + 2 s. A hyperbolic secant adiabatic inversion pulse was used with a bandwidth of 20 kHz for the FAIR labelling pulses (Wells et al., 2012). T1 maps were also acquired using an inversion recovery SE-EPI sequence for cerebral blood flow (CBF) quantification. The splenium of the corpus callosum was used as a landmark for consistency of slice positioning between subjects. CBF and arterial transit time (δa) maps were generated by fitting the ASL images and T1 maps to the model described by Buxton et al. (Buxton et al., 1998). ROIs were manually drawn in the cortex, hippocampus and thalamus across two 1 mm thick slices matching the regions that underwent histological analysis of NFT density (Fig. 1(I)). A ROI across the two most rostral slices in the frontal cortex was also drawn (where hyper-perfusion of the rTg4510 brains was particularly apparent from visual inspection).

### Diffusion tensor imaging (DTI)

A four shot Spin Echo EPI sequence was used to acquire sixteen slices. The olfactory bulbs were used as an anatomical landmark to maintain consistency in slice positioning between animals. The FOV was 20 × 20 mm with a matrix size of 100 × 100 and a slice thickness of 0.5 mm. Diffusion gradients were applied in thirty directions with the following parameters G = 0.25 T/m, Δ = 9.3 ms, δ = 5.5 ms, and b = 1050 s/mm^2^ to generate diffusion weighted images in addition to a single unweighted B0 image. Acquisition of 5 averages with a TR of 2000 ms gave a total imaging time of 43 min. Software written in Matlab was used to construct tensors at each voxel through a least squares solution approach (Batchelor et al., 2003). The parameters MD, FA as well as radial and axial diffusivity were calculated from the tensors following standard methods (Le Bihan et al., 2001, Song et al., 2002). Three slices posterior to the bregma were selected in the unweighted images and ROIs were manually drawn in the corpus callosum and grey matter areas of the hippocampus, cortex and thalamus (Fig. 1(I)). It should be noted that unlike ASL and CEST techniques (which suffer from relatively low SNR), DTI data were acquired with sufficient spatial resolution to resolve the corpus callosum and hence only DTI parameters from the white matter are reported. Furthermore, radial and axial diffusivity were only calculated in white matter structures. Mean values were calculated for the ROIs in each group for comparison of the DTI parameters.

### Chemical exchange saturation transfer (CEST)

The CEST sequence was acquired using a single slice gradient echo imaging sequence (TR = 6.1 ms, TE = 2 ms, flip = 5°, FOV = 20 × 20 mm2, slice thickness 3 mm, matrix size = 64 × 64). Saturation pulses were applied at 79 frequency offsets covering ± 6 ppm to encompass amide proton transfer (APT) saturation peaks around + 3.5 ppm. A reference offset at 200 ppm was also acquired for normalization. Shimming was performed across the entire imaging slice (3 mm thickness). ATP was calculated as the area under the MTRasym curves between 3.3 and 3.7 ppm on a pixel-by-pixel basis by fitting a polynomial function to Z spectra, correcting for off-resonance effects by cubic spline interpolation and subtracting the signal intensities at either side of the direct water saturation peak (van Zijl and Yadav, 2011b). ROIs were manually drawn in the cortex, hippocampus and thalamus.

### Glucose chemical exchange saturation transfer (glucoCEST)

CEST measurements (using identical parameters described above), were applied at baseline and then every 8 min following an intraperitoneal injection of 1 g/kg glucose for a total of 100 min following glucose delivery. To measure the in-vivo glucose uptake the chemical exchange contaminating effects are removed by subtracting pre-injection from post-injection and measuring all of the area under the MTRasym curve (Chan et al., 2012, Nasrallah et al., 2013, Walker-Samuel et al., 2013). ROIs were taken in the thalamus, cortex and hippocampus to match the regions that underwent histological analysis of NFT density (see Fig. 1(I)).

### Perfuse fixation

Following *in vivo* imaging, animals were terminally anaesthetised with Euthanal (0.1 mL) administered via intraperitoneal injection. The thoracic cavities were opened and the animals perfused through the left ventricle with 15–20 mL of saline (0.9%) followed by 50 mL of buffered formal saline at a flow rate of 3 mL per minute. Following perfusion, the animal was decapitated, defleshed, and the lower jaw removed. All brains were stored in-skull at 4 °C before being dispatched for histology.

### Histology and immunohistochemistry

Brain samples were then processed using the Tissue TEK® VIP processor (GMI Inc, MN USA). After processing, sections were embedded in paraffin wax to allow coronal brain sections to be cut. Serial sections (6-8 mm) were taken from using HM 200 and HM 355 (Thermo Scientific Microm, Germany) rotary microtomes.

Immunohistochemistry (IHC) was performed using a primary antibody for tau phosphorylated at serine 409 (PG-5; 1:500 from Peter Davies, Albert Einstein College of Medicine, NY, USA). Secondary antibody was applied and slides were then incubated with avidin biotin complex (ABC) reagent for 5 min (M.O.M. kit PK-2200, Elite ABC rabbit kit PK-6101, or Elite RTU ABC PK-7100 Vector Labs). After rinsing, slides were treated with the chromogen 3,3′-diaminobenzidine (DAB; Vector Laboratories, SK-4100) to allow visualisation. The slides were then cover slipped, dried and digitised using an Aperio Scanscope XT (Aperio Technologies Inc., CA, USA).

### *Ex vivo* image analysis and quantification

Images were viewed and analysed with Aperio ImageScope software (version 10.2.2319). In each study, two sections for each mouse were analysed. For each section stained, areas of specific interest (in this case the cortex, hippocampus and thalamus) were delineated in the same way as they were for the MR imaging and their area ascertained in mm^2^. Within these areas of interest, the number density of PG5 immunoreactivity was quantified using AperioImageScope.

In order to investigate the relationship between histological measurements of tau load and quantitative MRI, we performed two levels of correlation analysis. Firstly, for each region, NFT density was correlated to each of the MR metrics across the 9 rTg4510 subjects using Pearson's correlation. However, given limited evidence for within region correlations, we additionally performed a between region, ranked correlation analysis. rTg4510 brain regions were ranked as 0 (wildtype control mice), low (thalamus of rTg4510 mice) and high (cortex and hippocampus of rTg4510 mice) tau burden. For each of the regions, the percentage change in each of the MR parameters in each of the individual animals was calculated relative to the mean value of the WT controls, in order to account for intrinsic regional differences. Correlations in % change in CBF, MD, FA, CEST (from second cohort) against ranked tau burden (assigned 0 (WT), 1 (low) and 2 (high)) were investigated using Spearman's rank correlation coefficient. It should be noted that correlation of DTI metrics in the white matter to histology was not performed as there is no PG5 positive cell staining detected in the corpus callosum.

### Measurement of blood pressure and heart rate in awake and anaesthetised conditions

Measurements of heart rate and blood pressure were performed in a separate cohort of rTg4510 and WT control mice in awake and anaesthetised conditions. The primary motivation for acquiring these data followed the observation of marked hyperperfusion in the rTg4510 mice (see Results), a somewhat counter intuitive finding. Specifically we were interested to investigate whether increased CBF in the rTg4510 cohort could be partially explained by elevated blood pressure and heart rate under isoflurane anaesthesia compared to the WT controls. The differences in mean arterial pressure (MAP) and heart rate between WT rTg4510 mice studied under both conscious and anaesthetized conditions were carried out on animals purchased from Taconic with surgically-implanted telemetry devices. Conscious telemetry collections were performed continuously from all (non-anaesthetized) animals for 25 h to assess mean arterial pressure and heart rate. The mean cardiovascular data for each animal was calculated for 1-hour averages from the logging period data. To assess unconscious parameters, the animals were anaesthetized by placing the animal in a nose cone connected to a steady flow of 1.5% isofluorane and oxygen at 1 L/min, warmed with a water blanket maintained at 37 °C, and maintained under anaesthesia for approximately 1.5–2 h. Unconscious telemetry data were collected continuously starting immediately after and for the duration of anaesthesia. The mean arterial pressure and heart rate for each animal was calculated for 15-minute averages from the logging period data.

## Results

### High resolution structural MRI

High resolution (150 μm^3^) T2-weighted structural imaging was applied to investigate the morphometric phenotype of the rTg4510 model in comparison to WT controls. Measures of macroscopic brain atrophy confirmed regional brain shrinkage reflecting elevated NFT deposits. Manual segmentation of the hippocampus showed a significant difference between transgenic and wildtype animals (normalising to total intracranial volume (TIV) p = 2 × 10^− 11^, effect size = 5.5 (Fig. 1G)).

The TBM method allowed second-order voxel-wise group differences in brain atrophy to be elucidated, where gross differences in brain size are corrected for in the image alignment and normalisation procedure. Fig. 1(A–F) shows representative slices through the group average image with TBM statistics overlaid. Extensive bilateral changes throughout the transgenic brain relative to the group average were observed, including substantial atrophy of the striatum and dilation of the lateral, third and fourth ventricles. TBM also detected significant bilateral hippocampal atrophy and local contraction within the cortex and forebrain of the transgenic population.

### Quantitative MRI discriminates tau pathology

**Fig. 2:**
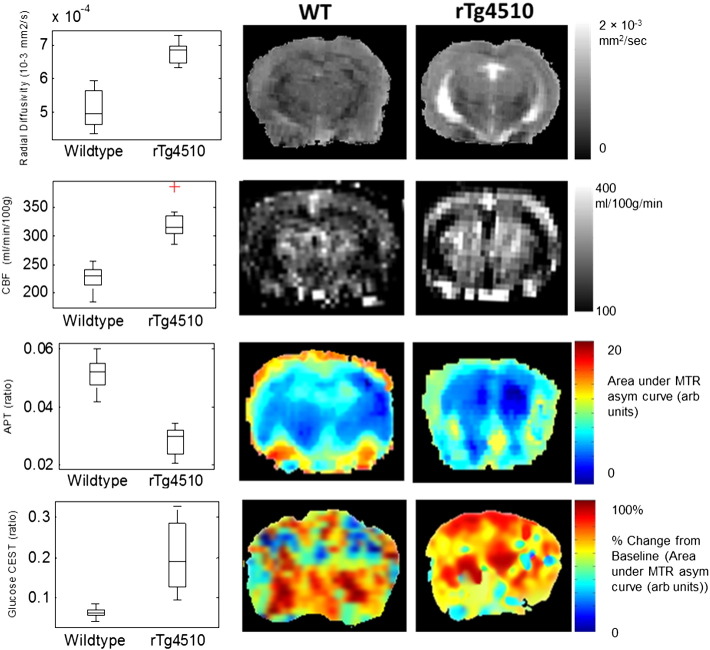
Quantitative MRI measurements that discriminate tau pathology from wildtype controls. 1st column: Box and whisker plots representing the median, interquartile range and range of the MRI parameters across the rTg4510 and WT mice with individual outliers highlighted by a red cross. 1st row: The radial diffusivity in the corpus callosum; 2nd row: The CBF in the frontal cortex; 3rd row: The CEST APT signal in the hippocampus; 4th row: glucoCEST signal in the cortex. 2nd/3rd column: Maps of radial diffusivity, CBF, CEST APT and GlucoCEST in a representative WT (2nd column) and rTg4510 (3rd column) mouse.

To investigate whether emerging quantitative MRI techniques could detect tau-driven differences in the rTg4510 model (relative to the WT controls), we applied ASL, CEST, DTI and glucoCEST to rTg4510 and WT control subjects. We observed a multi-parametric pattern of changes that differentiated the tau model from the healthy brain and were particularly pronounced in selective brain regions. Fig. 2 shows data from each technique in regions where complete discrimination was observed between the rTg4510 and WT subjects. The rTg4510 mice had increased radial diffusivity in the corpus callosum (p = 1.6 × 10^− 8^, effect size = 3.8), increased CBF in the frontal cortex (p = 7.4 × 10^− 10^, effect size = 3.8), decreased amide proton transfer (APT) in the hippocampus (p = 1.16 × 10^− 10^, effect size = 4.5) and increased glucoCEST signal in the cortex (p = 0.01, effect size = 2.6). Significant increases in CBF and MD were observed in the cortex, hippocampus and thalamus of the rTg4510 mice. Significantly reduced APT signal and increased MD and FA was observed in the cortex and hippocampus but no significant differences in these parameters were found in the thalamus. Increased glucoCEST was recorded in the cortex although no significant differences were detected in other regions. The raw CBF, APT, MD and FA values for each grey matter region are given in the supplementary material.

### Regional correlations of quantitative MRI to NFT density

**Fig. 3:**
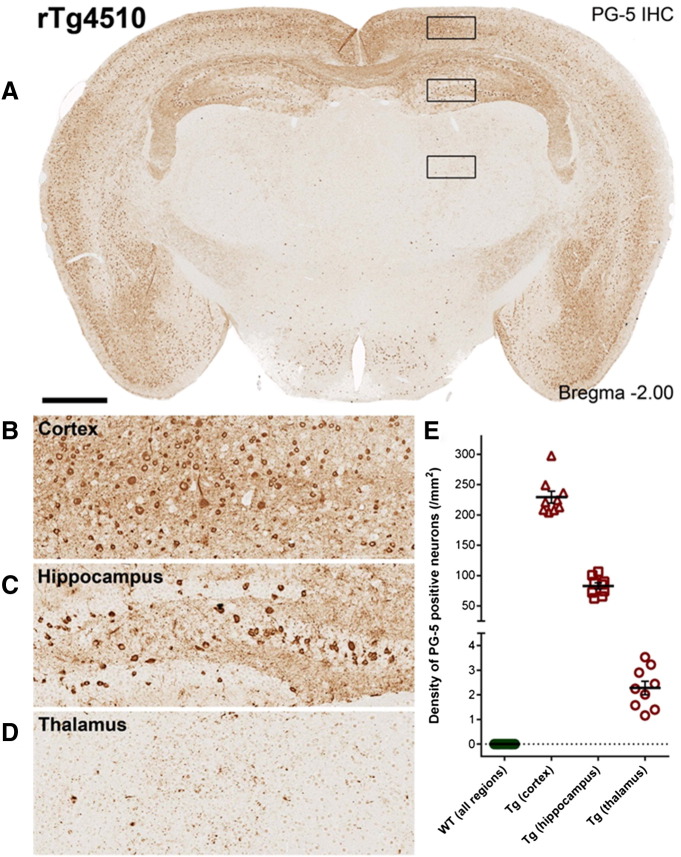
Immunohistochemistry to estimate regional PG-5 positive NFT density. A) Single slice from a representative rTg4510 mouse with staining for PG-5 positive NFTs. Marked regional dependence of NFT density is observable (see inset (B–D) for examples of cortical, hippocampal and thalamic NFT distribution). E) Quantitative regional estimates of NFT density for each of the 17 WT and 9 rTg4510 mice that underwent MRI (8.5 month age).

**Fig. 4:**
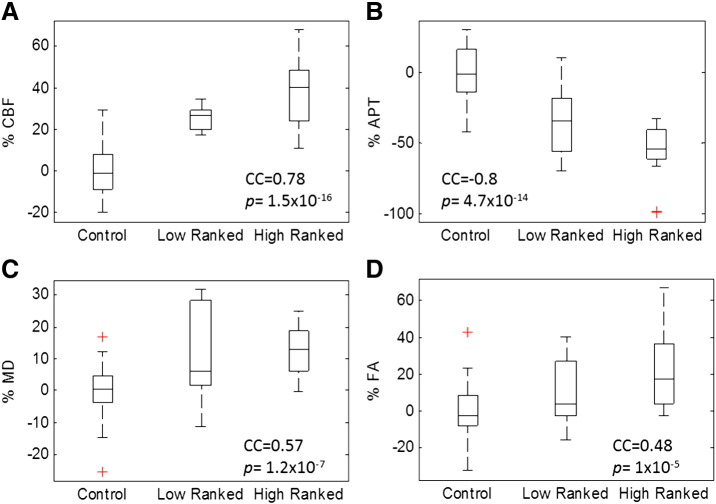
Quantitative MRI correlates to histological ranking of PG-5 positive NFT density. Percentage difference (normalised to control) in A) CBF, B) APT ratio C) MD and D) FA as a function of histological ranking of PG-5 positive NFT density. Spearman's non-parametric correlation coefficient was used to investigate a possible correlation of quantitative MRI to histological ranking (p-value and correlation coefficient (CC) reported in figure inset). Boxplots represent median, interquartile range and range of percentage difference in quantitative MR estimates from WT control with individual outliers highlighted by a red cross.

Immunohistochemistry was performed on each of the individual rTg4510 (n = 9) and WT (n = 17) control animals to estimate the density of PG-5 positive NFTs in cortical, hippocampal and thalamic regions (Fig. 3). No PG-5 positive cells were observed in any of the control mice and quantitative measures were made in the hippocampus, cortex and thalamus for each rTg4510 mouse. We observed a significant correlation between hippocampal volume and NFT density (Pearson's correlation coefficient (CC) = 0.81, p = 0.01) (Fig. 1H). In contrast, we found no correlation between cortical volume and cortical NFT density (Pearson's CC = 0.05, p = 0.58), despite histology showing the accumulation of NFTs in both hippocampus and cortex. Indeed, limited within region correlations were observed using CBF, DTI and CEST based metrics. Therefore, a secondary correlation analysis was performed in which the cortex and hippocampus were ranked as “high” pathological tau burden (mean NFT density = 229 cells/mm^2^ and 83 cells/mm^2^ respectively) and the thalamus as “low” tau burden (mean NFT density 2.3 cells/mm^2^) (Fig. 3). When we examined the relationship between percentage difference in MRI parameters from WT controls (ranked as 0 tau burden) and tau density, we observed a significant correlation of CBF, APT, FA and MD to the ranked data (Fig. 4). There was a positive correlation of CBF (Spearman's CC = 0.78, p = 1.5 × 10^− 16^), FA (Spearman's CC = 0.48, p = 1 × 10^− 5^) and MD (Spearman's CC = 0.57, p = 1.2 × 10^− 7^) to group ranked tau density with CEST displaying a negative relationship (Spearman's CC = 0.8, p = 4.7 × 10^− 14^). However, similarly to the structural measurements, there was no statistical difference in the percentage difference from control between the cortex and hippocampus (the “high” ranked regions) in each of the MR measures, despite the cortex presenting with a threefold increase in NFT density relative to the hippocampus. This finding suggests a monotonic relationship exists between measures of CBF, APT, MD, FA and NFT density within a degree of tau burden which dissociates between regions of “high” tau burden (the cortex and hippocampus) in the 8.5 month rTg4510 mice.

### CBF and MD increases in the thalamus

**Fig. 5:**
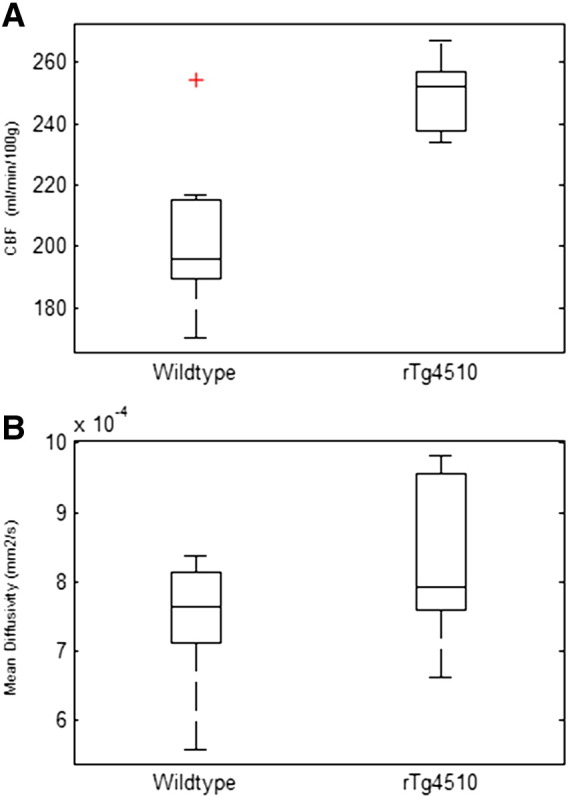
CBF and MD in the WT and rTg4510 thalamus (region of “low” tau density). ASL CBF (A) and MD (B) estimates in the WT and rTg4510 thalamus (region of “low” tau density). Boxplots represent median, interquartile range and range with individual outliers highlighted by a red cross.

The thalamus presented with relatively “low” NFT density at this time point (2.3 cells/mm^2^) and no significant atrophy was detected with TBM (see above). Yet, we observed increased CBF (p = 3 × 10^− 6^) and MD (p = 0.04) in the thalamus of the rTg4510 cohort relative to WT control mice (Fig. 5). Whilst the interpretation of these data is complex due to the more severe pathology in the surrounding, functionally connected, regions (see Discussion), this does provide some encouragement that such techniques may provide sensitivity to more subtle tau-induced pathology that proceed gross morphometric changes.

### Diffusion tensor imaging of white matter

**Fig. 6:**
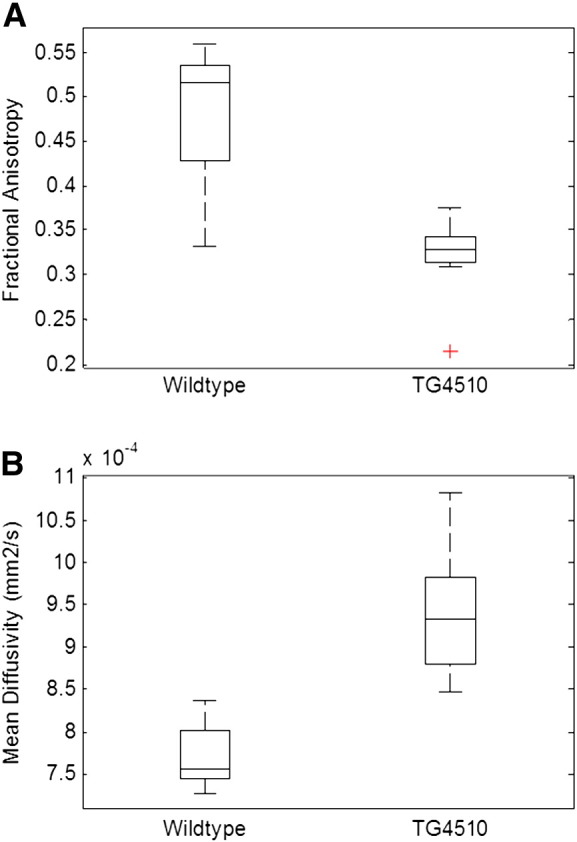
Diffusion tensor imaging measurements in the white matter (corpus callosum) of rTg4510 and WT controls. DTI measurements in the white matter (corpus callosum) of rTg4510 and WT controls. Boxplots represent median, interquartile range and range with individual outliers highlighted by a red cross.

DTI was employed to investigate microstructural changes in the white matter of the rTg4510 mice relative to the normal brain. The mean ROI measurements of FA and MD in the corpus callosum showed significant differences between the transgenic and control groups (Fig. 6). The transgenic group exhibited a reduced FA of 0.32 ± 0.05 compared to the controls (0.48 ± 0.07, p = 4.5 × 10^− 6^, effect size = 2.7). In contrast, the MD of 9.4 ± 0.8 × 10^− 4^ mm^2^/s observed in the transgenic group was greater than in the controls (7.7 ± 0.4 × 10^− 4^ mm^2^/s, p = 6.3 × 10^− 8^, effect size = 3). Most marked was radial diffusivity, in which we saw a clear discrimination between the wildtype and the transgenic groups (p = 1.5 × 10^− 8^, effect size = 3.8) (Fig. 2, first row). This separation did not occur in the axial diffusivity values where there was no significant difference between groups (rTg4510 = 11.1 ± 0.9 × 10^− 4^ mm^2^/s, control = 10.7 ± 0.9 × 10^− 4^ mm^2^/s, p = 0.36).

### Blood pressure and heart rate under awake and anaesthetised conditions

rTg4510 mice had higher mean arterial pressure (MAP) and heart rate compared to the wild type mice during the active 12-hour dark phase of the 24-hour conscious study interval. The average difference in MAP between groups was 17.4 mm Hg, with a maximum difference at the 9th hour of 24.1 mm Hg ± 5.5 and a minimum difference at the 12th hour of 9.0 mm Hg ± 6.5. The average difference in the heart rate between groups was 142 bpm, with a maximum difference at the 6th hour of 183 bpm ± 27 and a minimum difference at the 12th hour of 68 bpm ± 24. No differences were noted in these parameters during the light cycles. In contrast, in the same mice anaesthetized for a 2 hour period under comparable conditions used in the MRI experiments, no significant differences in MAP or heart rate were measured between wildtype (WT) and rTg4510 groups.

## Discussion

This study reports the first application of non-invasive imaging biomarkers, sensitive to perfusion, protein accumulation, glucose uptake and tissue microstructure to generate a multi-parametric tissue characterisation of tau pathology. We have demonstrated the sensitivity of non-invasive imaging to provide brain tissue correlates of tau pathology in white matter and in regions of high NFT density in the grey matter. Hyperperfusion and increased MD were observed in a region of low NFT density with no detectable structural changes. This finding demonstrates the value of ASL and DTI for the identification of tau-driven pathological processes prior to marked atrophy, suggesting their utility as biomarkers for the early assessment of novel tau therapeutics.

Histological measures of pathological tau burden in the rTg4510 mice demonstrated a strong regional dependence of NFT density ranging from low (2.3 counts/mm^2^ in the thalamus) to high (229 counts/mm^2^ in the cortex) (Fig. 3). This is to be expected as expression of tau is driven by a forebrain specific promoter (CaMKII) in the rTg4510 mouse (Ramsden et al., 2005). We sought to exploit this marked dynamic range of NFT density (as quantified using PG-5, pS409 tau) between regions to investigate correlations between ranked tau burden and difference in MR parameters from control (to account for intrinsic regional differences). We observed significant correlations between the percentage change from control in CBF, MD, FA and CEST across regions free of NFTs (wildtype), low NFT density (rTg4510 thalamus) and high NFT density (rTg4510 cortex and hippocampus), indicating a monotonic relationship between NFT density and quantitative MR changes between different regions of varying tau load at this time point. This observation provides encouragement that multi-parametric quantitative MRI could be used to broadly classify regions of negligible, low and high NFT density for non-invasive staging of AD. The percentage change in MR parameters in rTg4510 from control were very similar between the cortex and hippocampus (high ranked data), despite the marked difference in the overall NFT density between these two brain regions. This may be a feature of the rTg4510 model at this time point or reflect the greater and more selective vulnerability (or different inherent composition) of the hippocampus, a region known to present with marked abnormality in AD patients. Manual volumetric estimates of the hippocampus showed the strongest within-region correlation to NFT density (Fig. 1) indicating a strong link of NFT formation and hippocampal atrophy at this time point. We observed limited within-region correlations of NFT density to other quantitative MR parameters which may be due to the relatively narrow distribution of NFT density within each region (Cortex: 229 (± 28), Hippocampus: 82.9 (± 15), Thalamus: 2.3 (± 0.8)) at this time point. Longitudinal characterisation of this model using multiple quantitative MRI parameters would be valuable to understand the regional nature of the temporal progression of changes across the individual imaging modalities. It could be that the relationship between PG-5 positive NFT density and individual MRI parameters would be stronger at particular stages in the progression of tau pathology (for example as tangles begin to form in the model at 4–6 months of age). It should be noted that a limitation of this study is the division of the applied techniques into two separate cohorts of rTg4510 and control mice of subtlety different ages (8.5 and 9.5 months). Structural imaging, ASL and DTI were acquired in the first cohort (imaging time 2 h, 45 min) and Glucose CEST and CEST were acquired in the second cohort (imaging time ~ 1 h, 40 min). Furthermore, the relatively late time point (8.5–9.5 months) may restrict evidence for specificity of MR parameters to abnormal tau within the high ranked regions, as marked neurodegeneration is likely to have already occurred. Future application of these techniques at earlier time-points in the rTg4510 and alternative mouse models of AD may help elude a multi-parametric MRI signature with high specificity to tau pathology.

Structural MRI has already established itself as a reliable biomarker in the clinical diagnosis of Alzheimer's disease (Dubois et al., 2007, Frisoni et al., 2010, Jack et al., 1997); characteristic grey matter reduction and ventricular enlargement can be visualised using standard T1-weighted sequences. As well as revealing signs of atrophy, which can inform differential diagnosis of the disease, antemortem MRI has previously been found to correlate well with measures of cognitive deterioration and post-mortem Braak staging of the disease (Csernansky et al., 2004, Jack et al., 2002, Vemuri et al., 2008, Whitwell et al., 2008). The pattern of brain atrophy is closely related to regional NFT accumulation found in AD patients (and quite different to the pattern of amyloid plaques) (Braak and Braak, 1991, McDonald et al., 2009), and thus atrophy remains a valuable biomarker in the evaluation of tau based therapeutics. The rTg4510 mouse mimics the features of clinical neurodegenerative diseases such as Alzheimer's disease with cognitive deficits, NFT formation and neuronal loss in the forebrain and hippocampus (Ramsden et al., 2005, Santacruz et al., 2005). Previously published work has reported structural changes in rTg4510 mice in the hippocampus and cerebral cortex, as well as enlargement of the ventricles using *in vivo* MRI (Yang et al., 2011). Using TBM, we were able to extend these observations and additionally detect local volume reductions in the cortex, hippocampus, caudate putamen (striatum) and olfactory bulbs as well as ventricular expansion (Fig. 1A–F). These morphometric changes coincide with NFT pathology, thereby supporting the role of tau in neurodegeneration. At this time point, the transgenic brains have undergone gross atrophy which is noticeable by eye in *in*-*vivo* structural scans. We therefore anticipate that TBM will most valuable in the detection of subtle structural abnormalities that occur at earlier stages of tauopathy. The negative correlation that we measured between hippocampal volume and NFT density provides evidence of a direct relationship between NFT pathology and atrophy, whilst highlighting the sensitivity of *in*-*vivo* MRI to subtle volume changes. This correlation was not present in the cortex, despite higher NFT density. This may be because the hippocampus has a more rapid progression of tangle formation and thus the subsequent neurodegeneration is more advanced at 8–9 months of age.

AD patients and subjects in prodromal disease stages present with regionally dependent hypo- and hyper-perfusion (Wolk and Detre, 2012) compared to healthy control subjects. In this work we have observed increased CBF in the rTg4510 mice, which correlated with ranking of tau burden across different brain regions (Fig. 4). There is a paucity of studies that have applied ASL to mouse models of AD, although cortical hypo-perfusion has been reported in two amyloid models (Faure et al., 2011, Weidensteiner et al., 2009). Our contrasting result (when applying ASL to a mouse model of tau pathology for the first time) may give clues into the different mechanisms underlying regional changes in CBF in AD. The increased CBF may reflect aberrant neuronal excitability (Palop et al., 2007), neural compensatory mechanisms (Wierenga et al., 2011) or a breakdown of auto-regulation, though we do not have evidence that alludes to one mechanism at this point. The similarity of the blood pressure measurements in the anaesthetised rTg4510 and wildtype animals indicates that systemic differences in BP are not responsible for the elevated CBF in the rTg4510 animals, and that the observed hyper-perfusion is brain specific.

A number of clinical studies using DTI have observed increased MD and reduced FA in the white matter of the brains of AD patients when compared to healthy controls (Jack, 2012, Sabbagh et al., 2013). Our findings of increased MD and reduced FA in rTg4510 mice relative to wild type controls are consistent with this and suggest that the aberrant white matter microstructure observed in AD may be, at least in part, a consequence of the tau pathology underlying the disease. Our observation of a significant increase in radial diffusivity in the presence of unchanged axial diffusivity in the corpus callosum of the rTg4510 mice is consistent with this and suggests a reduction in myelination, mirroring previous observations in sub regions of the corpus callosum in AD sufferers (Di Paola et al., 2010). These findings are highly concordant with an earlier longitudinal study that applied DTI methods to investigate the white matter of the rTg4510 model (Sahara et al., 2014). Electron microscopy measures were performed and demonstrated the presence of tau inclusions in the white matter and subsequent micro-structural disorganisation relative to WT controls at later time points (Sahara et al., 2014). The effects of amyloid plaques, the other hallmark pathology of AD, have been investigated in previous studies (Song et al., 2004, Sun et al., 2005) where differences in DTI parameters have been observed at late time points after the appearance of amyloid plaques. Abnormal myelination patterns have been identified prior to appearance of NFTs and amyloid plaques in a mouse model exhibiting both pathologies (Desai et al., 2009). The MD measurements in grey matter regions are in good agreement with previous studies that have detected elevated values in the brains of AD patients (Fellgiebel et al., 2004, Kantarci et al., 2001, Rose et al., 2008) and in mouse models exhibiting amyloid pathology at late stages of progression (Sun et al., 2005). The positive correlation between the increased MD and the tau density ranking suggest that the disruption to the cytoarchitecture indicated by these increases may be driven by the pathology. Furthermore, the positive correlation of FA with tau density may indicate that the structural reorganisation that occurs may have directional specificity. FA has been found to be reduced in AD patients in the hippocampus (Fellgiebel et al., 2004) and the thalamus (Rose et al., 2008) which contradicts the findings in this study where the FA was increased significantly in the cortex and hippocampus in the transgenic mice and there is no significant difference in the thalamus. These differences may be due to the addition of amyloid-beta pathology present in the brains of AD patients or the inherent differences in murine grey matter tissue structure.

Magnetisation transfer ratio has been studied in clinical Alzheimer's disease (Hentschel et al., 2004, van Es et al., 2006). CEST MRI differs from magnetization transfer studies by probing the chemical exchange in mobile compounds rather than semisolids (van Jijl and Yadav, 2001). Contrast in CEST APT-weighted imaging is provided by the exchange of amide protons in mobile cellular proteins and peptides with water protons. The reduction in APT in the rTg4510 animals may indicate a break down in normal chemical exchange caused by tau pathology. However the differences in APT may also be influenced by pH and the longitudinal relaxation time (T1), which may change due to cell death, for example (van Zijl and Yadav, 2011), meaning that as a biomarker, CEST distinguishes normal controls from rTg4510 mice but does so in a non-specific manner.

GlucoCEST was also able to discriminate between tau pathology and normal tissue. Given its reliance on the injection of normal, unlabelled glucose, this novel technique has potential for translation into a clinical setting to diagnose and track the progression of neuro-degeneration, without the associated exposure to radiotracers required for PET imaging. In this work we observed an increased glucoCEST signal in the cortex of the rTg4510 mice relative to WT controls. This finding is supported by a recent study that reported increased glucose utilisation and hyper-metabolism in the cortex in the same mouse model (Nilsen et al., 2013). However, the glucoCEST signal arising from free glucose and products arising from its metabolism can be present in any of three tissue compartments: intravascular, extravascular–extracellular, and intracellular (Chan et al., 2012, Nasrallah et al., 2013, Walker-Samuel et al., 2013). The increased cortical glucoCEST signal that we measured in the rTg4510s could therefore be caused by increased accumulation of glucose in the tissue due to a reduction in glucose metabolism alongside an increased delivery due to the elevated CBF to the cortical regions. The technique's ability to clearly distinguish tau pathology is the first application to neuro-degeneration and provides further insight into the metabolic processes of the tau pathology.

The thalamus of the rTg4510 mice was found to have relatively low NFT density (mean = 2.3 cells/mm^2^) with no detectable atrophy (based on TBM), mirroring human tissue at early Braak staging with low NFT density (Whitwell et al., 2008). We observed an increased CBF and MD in this region relative to WT controls. This finding suggests that microstructural and haemodynamic changes that occur without gross neuronal loss can be detected in this brain region with MRI and highlights the value of multi-parametric measures in the same subject for sensitive detection of tau pathology. These imaging changes may reflect very early local markers of tau pathology in the thalamus, but we cannot rule out that some of these differences may also be secondary to the large tau pathology in the forebrain structures, many of which are known to project to the thalamus. This may be important as we know that tau aggregates can transfer and propagate/spread within synaptic circuits (Clavaguera et al., 2009, de Calignon et al., 2012). There is post-mortem evidence in AD for this regional spread (Zhou Gennatas et al., 2012) and more recent neuroimaging studies in human AD suggest “epicentres” (Zhou et al., 2012) or “nodes” (Raj et al., 2012) that may link to network dysfunction.

In conclusion, this study demonstrates the value of non-invasive multi-parametric quantitative MRI for sensitive detection of tau pathology in the rTg4510 model of AD. These novel data represent a platform for future longitudinal and therapeutic efficacy studies in this model. The 5-tiered measurement approach is directly translatable and merits application in clinical populations to investigate the pattern of MR changes in regions known to follow the well-defined stages of NFT progression. Estimates of perfusion and diffusion can detect changes in healthy tissue in a region of low NFT density (2.3 cells/mm^2^) with no detectable atrophy which provides promise for the use of these markers to identify and track subjects early in the Alzheimer's disease continuum.

## Funding

JW is supported by the Medical Research Council (MR/J013110/1). SWS is supported by the Wellcome Trust. HEH is supported by the NC3Rs (NC/K500276/1)SO receives funding from the EPSRC (EP/H046410/1, EP/J020990/1, EP/K005278), the MRC (MR/J01107X/1), the EU-FP7 project VPH-DARE@IT (FP7-ICT-2011-9-601055), the NIHR Biomedical Research Unit (Dementia) at UCL and the National Institute for Health Research University College London Hospitals Biomedical Research Centre (NIHR BRC UCLH/UCL High Impact Initiative - BW.mn.BRC10269). M M is supported by the UCL Leonard Wolfson Experimental Neurology Centre (PR/ylr/18575). MJ C receives funding from EPSRC (EP/H046410/1).NMP is supported by the MRC (MR/G0900207-3/1). JO'C is supported by the MRC (MR/J500422/1). ML receives funding from Medical Research Council (MR/J013110/1); the King's College London and UCL Comprehensive Cancer Imaging Centre CR-UK & EPSRC, in association with the MRC and DoH (England); the National Centre for the Replacement, Reduction and Refinement of Animal in Research (NC3Rs); UK Regenerative Medicine Platform Safety Hub (MRC: MR/K026739/1);NC and OI are supported by Eli Lilly and Company.

## Appendix A Supplementary data

Supplementary Results.
