# Supplementary material for: In vivo imaging of tau pathology using multi-parametric quantitative MRI
Source: Neuroimage. 2015 May 1;111:369–78. doi: 10.1016/j.neuroimage.2015.02.023 (PMC4626540; doi:10.1016/j.neuroimage.2015.02.023)
Supplement: Supplementary file 1 — Supplementary Results. [file mmc1.docx]

## **Results (Supplementary Material)**

**Arterial Spin Labelling to Estimate Cerebral Blood Flow**

The CBF was significantly greater in the TG4510 group in comparison to litter matched controls (p<0.05) in each of the cortical, hippocampal and thalamic regions. The mean CBF (SD) for the TG4510 and control animals was: cortex: 291 (27), 217 (31); hippocampus: 283 (38), 199 (20.6); thalamus: 248 (23), 199 (24.9) ml/100g/min respectively. The CBF in the frontal cortex showed complete discrimination between the TG4510 and control animals (Figure 2) with a mean of 321(30) and 226 (20) respectively. The mean arterial transit time (δa) (SD) for the TG4510 and control mice was: cortex: 0.37 (0.06), 0.36 (0.05); hippocampus: 0.31 (0.03). 0.36 (0.05); thalamus 0.27 (0.03), 0.33 (0.05);frontal cortex 0.29 (0.05), 0.29 (0.04) seconds respectively. The arterial transit time was significantly reduced in the hippocampus and thalamus of the TG4510 mice in comparison to the WT controls.

## **DTI in the Grey Matter**

The ROIs in the cortex and hippocampus, areas of high density of tau pathology, showed increased FA and MD in the transgenic group. In the cortex, the FA in the transgenic and wild type groups were 0.18±0.03 and 0.15±0.01 and the MD was 8.24±0.49x10^-4^ mm^2^/s and 7.64±0.35x10^-4^ mm^2^/s respectively. These differences were statistically significant (p<0.05). The Hippocampus measurements of FA were 0.21±0.04 and 0.17±0.03 for the transgenic and wildtype groups respectively and the MD values were 9±0.49x10^-4^ mm^2^/s and 7.7±0.59x10^-4^ mm^2^/s, only the MD and FA group differences were significant.

**CEST to Estimate Amide-Water Proton Transfer**

The apt CEST results show a statistically significant decrease in the chemical exchange in the Cortex and Hippocampal regions and no significant difference in the Thalamus. The mean and standard deviation (SD) for each region in the wild type and TG4510 consecutively is: Cortex mean 0.11±0.022, mean .042±0.025; Hippocampal mean 0.053 ±0.007, mean 0.026±0.005; Thalamus mean 0.96 ± 0.024, mean 0.73±0.023 APTasym at 3.5 ppm.

**GlucoCEST to estimate glucose uptake**

Over the complete time course of 100 minutes from bolus injection the average glucoCEST enhancement in the cortex of the TG4510 group (0.21± 0.11) was statistically significantly (p<0.05) higher than the average signal of the wild type group (0.07 ±0.04).The thalamus mean TG4510 (0.24± 0.08) and wild type (0.25 ± 0.05); hippocampus TG4510 (0.13±0.05) and wild type (0.07 ±0.04) and were not significantly different.
